# Multiple Localization Analysis of the Major QTL—*sfw 2.2* for Controlling Single Fruit Weight Traits in Melon Based on SLAF Sequencing

**DOI:** 10.3390/genes15091138

**Published:** 2024-08-28

**Authors:** Yi Cai, Di Wang, Ye Che, Ling Wang, Fan Zhang, Tai Liu, Yunyan Sheng

**Affiliations:** 1 Horticulture and Landscape Department, Heilongjiang Bayi Agriculture University, Daqing 163000, China; c1299234359@163.com (Y.C.); wlbynd@163.com (L.W.); zhangfan2434@126.com (F.Z.); 2 Horticultural Branch of Heilongjiang Academy of Agricultural Sciences, Harbin 150069, China; 3 Daqing Branch of Heilongjiang Academy of Agricultural Sciences, Daqing 163000, China; dqnkywd@126.com (D.W.); cheye13836789851@163.com (Y.C.); liutai15663669385@163.com (T.L.)

**Keywords:** melon, single fruit weight, SLAF sequencing, QTL localization

## Abstract

*Cucumis melo* is an annual dicotyledonous trailing herb. It is fruity, cool, and refreshing to eat and is widely loved by consumers worldwide. The single fruit weight is an important factor affecting the yield, and thus the income and economic benefits, of melon crops. In this study, to identify the main QTLs (quantitative trait locus) controlling the single fruit weight of melon and thereby identify candidate genes controlling this trait, specific-locus amplified fragment sequencing (SLAF) analysis was performed on the offspring of female 1244 plants crossed with male MS-5 plants. A total of 115 individual plants in the melon F_2_ population were analyzed to construct a genetic linkage map with a total map distance of 1383.88 cM by the group in the early stages of the project, which was divided into 12 linkage groups with a total of 10,596 SLAF markers spaced at an average genetic distance of 0.13 cM. A total of six QTLs controlling single fruit weight (*sfw* loci) were detected. Seven pairs of markers with polymorphisms were obtained by screening candidate intervals from the SLAF data. The primary QTL *sfw2.2* was further studied in 300 F_2:3_ family lines grown in 2020 and 2021, respectively, a positioning *sfw2.2* between the markers CY Indel 11 and CY Indel 16, between 18,568,142 and 18,704,724 on chromosome 2. This interval contained 136.58 kb and included three genes with functional annotations, *MELO3C029673*, *MELO3C029669*, and *MELO3C029674*. Gene expression information for different fruit development stages was obtained from 1244 and MS-5 fruits on the 15d, 25d, and 35d after pollination, and qRT-PCR (quantitative reverse transcription–PCR) indicated that the expression of the *MELO3C029669* gene significantly differed between the parents during the three periods. The gene sequences between the parents of MELO3C029669 were analyzed and compared, a base mutation was found to occur in the intronic interval between the parents of the gene, from A-G. Phylogenetic evolutionary tree analysis revealed that the candidate gene *MELO3C029669* is most closely related to *Pisum sativum Fimbrin-5 variant 2* and most distantly related to *Cucumis melo* var. *makuwa*. Therefore, it was hypothesized that *MELO3C029669* is the primary major locus controlling single fruit weight in melon. These results not only provide a theoretical basis for further studies to find genes with functions in melon single fruit weight but also lay the foundation for accelerating breakthroughs and innovations in melon breeding.

## 1. Introduction

Melon is a major economic plant in the Cucurbitaceae family that is rich in a variety of nutrients and is widely cultivated worldwide. Single fruit weight is an important external quality trait of cucurbit crops that not only affects yield but also determines consumer acceptance. Therefore, studying single fruit weight is important in melon breeding programs. To date, many researchers have performed QTL localization and candidate gene identification related to controlling single fruit weight based on genetic populations and linkage maps [1,2,3]. Most QTLs related to single fruit weight have been detected in the studies of horticultural fruit-related traits [4,5,6,7,8,9,10,11,12,13], and these QTLs have been highly important for accelerating the breeding of horticultural crops.

Previously, QTLs associated with single fruit weight traits were identified in watermelon, pumpkin, and wax gourd. Guo et al. [14] constructed 100 recombinant inbred lines (RILs) using small-fruited watermelon sh14-11 and large-fruited watermelon N14 as parents. Using whole genome resequencing (WGR) technology, a high-quality genetic map was constructed, and a locus controlling FW was localized on chromosome 2, which had an LOD value of 3.21, a PVE of 15.135%, and an ADD of 0.246. The *FW9* locus was detected on chromosome 9 of the watermelon at 246.57–256.50 cM, with a logarithm of odds (LOD) value of 6.29 and a phenotypic contribution of 20.12% [15]. Li et al. [16] detected a QTL associated with fruit size on chromosome 8, which was named *fs-B171*. The locus was located at LG8 0–15.75 cM, with an LOD value of 8.03 and a phenotypic contribution of 41.37%, indicating a significant additive effect on fruit weight gain. Using sequencing technology, Han et al. [17] constructed a high-density genetic map of pumpkin containing 20 chromosomes and identified four QTLs associated with fruit weight, with LOD values ranging from 5.84 to 9.19 and phenotypic variation explained (PVE) values ranging from 4.01% to 5.13%. The colocalization of *fw12.1* and *fw16.1* with *scs12.1* and *fth16.1*, respectively, suggested that flesh thickness (FTH) and seed cavity size (SCS) have a certain genetic basis associated with fruit weight (FW), and the results of this study have provided an important theoretical basis for pumpkin quality improvement through breeding. In the genetic analysis and QTL localization of wax gourd fruit-related traits, two QTL loci, *fw3.1* and *fw6.1*, controlling fruit weight, were located in chr03 and chr06, respectively, with LOD values of 5.73 and 3.31. *fw3.1* and *fd3.1* controlling fruit width had similar genetic distances, which suggested that fruit weight and fruit width might be controlled by the same gene [18].

In recent years, melon has also shown extensive diversity in terms of fruit size, and there have been numerous reports on the use of QTL localization techniques for studying melon fruit size-related traits. A QTL *qFWT6* associated with single fruit weight trait in melon, located at 79.162 cM–79.412 cM on chromosome 6, with an additive effect of −21.85 and a phenotypic contribution of 9.53%, was reported in the study by Zhang et al. [19]. Amanullah et al. [20] identified 4 QTL associated with fruit weight at different positions on chromosomes 1, 9, and 12 of melon, namely *FWT1.1*, *FWT1.2*, *FWT9.1*, and *FWT12.1*, with LOD values ranging from 3.53 to 7.55. Among them, the additive effects of *FWT1.1* and *FWT12.1* were positive, whereas the *FWT1.2* and *FWT9.1* had negative additive effects. Monforte et al. [21] in a study on the genetic basis of fruit morphology in horticultural crops: insights from tomato and melon, and introduced four QTLs *FWMQ2*, *FWMQ3*, *FWMQ8*, and *FWMQ11* that control melon fruit weight, where the QTL *FWMQ2* is co-segregating with the gene *a*, which controls sex determination in female flowers. In a previous study by Monforte et al. [22], it was concluded that the loss of protease activity encoded by the gene results in plants with monoecious flowers and the presence of stamens in female flowers limits longitudinal ovary growth and reduces fruit elongation. Thus, the sexually expressed gene has an effect on fruit size. Díaz et al. [23] constructed an F_2_ population with the Indian wild melon variety “Trigonus” and the western melon variety “Piel de Sapo” as parents, and analyzed the QTLs controlling traits related to the domestication of fruit morphology. Ten QTLs associated with fruit size were identified, including those controlling fruit length and fruit weight all located on chromosomes 2, 4, 6, and 8, while those controlling fruit width were located only on chromosomes 4 and 8. Díaz et al. [24] also identified seven QTLs related to fruit size in another study, with five QTLs controlling fruit length, *flqs2.1*, *flqs3b.1*, *flqs6a.1*, *flqs8.1*, and *flqs10b.1*, respectively. With LOD values ranging from 2.92 to 16.85; and 2 QTLs controlling the width of fruits, *fdqs3a.1* and *fdqs12.1*, with LOD values of 25.6 and 52, respectively. Zhao et al. [25] detected QTL *fwqaz8.1* controlling the FW trait in the region of about 2.95–4.77 Mb on chromosome 8 in a cross between wild and cultivated melons. This region is consistent with the previously reported QTL *fwqc8.1*; this QTL negatively contributed to the increase in melon fruit weight. Campos et al. [26] constructed the introgression line (IL) using the wild germplasm Ames 24297 (TRI) and ‘Piel de Sapo’ (PS) as the parents, to study fruit-related traits and the degree of FW trait variation ranged from +42% (TRI05-2) to −57% (TRI04-3) compared to PS, with TRI05-2 being the only IL that increased FW. Major QTLs associated with single fruit weight on chromosomes 5 and 8 were identified by Pereira et al. [27]. F_2_ populations (200 plants) were generated from two varieties (snake and thick-skinned melon) as parents to locate and analyze QTLs controlling traits such as single fruit weight and fruit shape, and eight QTLs controlling single fruit weight, soluble solids, etc., were newly located. Of these, two primary QTLs controlling the traits of single fruit weight in melon were located on chromosome 8, with phenotypic contributions of 20.60% and 12.8%, respectively [28].

These previous studies have laid a theoretical foundation for in-depth research on the fine positioning of single fruit weight-related traits and the associated candidate genes in melon. Candidate genes associated with single fruit weight in melon were previously screened based on the candidate intervals located by QTLs, and single candidate genes associated with fruit size, Cm arY5 (axial regulator YABBY 5) and Cm Arf (auxin response factor), were identified on chromosome 5 and chromosome 11 of melon, respectively [29]. YABBY-like transcription factors are associated with the evolution of tomato fruit size during domestication, and growth factors determine final fruit size by controlling cell division and cell proliferation, suggesting that these two candidate genes may play important roles in determining melon fruit size. Pereira et al. [28] also identified the gene *MELO3C014402*, which encodes the protein FANTASTIC FOUR 2 and is associated with meristematic tissue development and cell size regulation; therefore, *MELO3C014402* is considered a potential gene related to single fruit weight. *FWQP2.2* is located on melon chromosome 2, coincident with the gene associated with the sex of female flowers in melon, suggesting that differences in fruit weight in melon populations may be the result of sex determination [30]. *MELO3C029669*, the candidate gene found in this study, is related to the filamentous protein fimbrin-5. Five filamentous protein-like genes, *FIM1-FIM5*, were detected by Zhang et al. [31] in an Arabidopsis genome study, of which *AtFIM4* and *AtFIM5* have been intensively studied. It is hypothesised that both *AtFIM4* and *AtFIM5* are expressed in pollen and act synergistically. *AtFIM5* regulates actin bundles throughout the pollen tube, and *AtFIM4* begins to be expressed only after the pollen grains are hydrated. When the expression of both genes is reduced at the same time, the extent of filamentous bundles in the pollen tubes of Arabidopsis decreases, and pod length decreases significantly, which affects the size of the plants. Therefore, based on previous research, *MELO3C029669*, which is related to the fiber bundle protein fimbrin-5, may be considered the main candidate gene for control of the single fruit weight trait in melon.

With the rapid development of technology, molecular marker analysis has been widely applied in various crop genetic breeding studies. The commonly used molecular markers include SSR (simple sequence repeat) markers, CAPS (cleaved amplified polymorphic sequences) markers, Indel (insertion-deletion) markers, and more than ten other types of markers. Xu et al. [32] constructed a genetic linkage map of pumpkin rootstock containing 15 linkage groups using 95 pairs of polymorphic SSR markers and detected 3 QTLs related to cold resistance. Zhang et al. [33] used CAPS marker technology to identify the *xa25* gene in rice; the CAPS marker designed for this study was isolated with the *xa25* gene to clearly differentiate the rice *xa25*/*Xa25* genotypes. Seo et al. [34] developed Indel markers for the development and validation of pod tolerance in soybeans. The marker successfully distinguished between pod-shattering-tolerant and pod-shattering-susceptible genotypes and can be effectively used for marker-assisted selection for pod resistance in soybean plants.

In this study, we selected wild-type melon (1244) females and thick-skinned melon (MS-5) males as test materials and obtained three segregating populations, F_1_, F_2_, and F_2:3,_ after formulating the hybrid combinations. Based on the genetic linkage map constructed from SLAF sequencing results about the F_2_ population [35], we obtained candidate intervals for the primary loci controlling single fruit weight in melon, and further localized the QTLs *sfw2.2* for the single fruit weight trait by using molecular markers to identify the linkage markers associated with the trait and shorten the candidate intervals. Moreover, the candidate genes *MELO3C029669* were identified. We also carried out qRT-PCR verification of the gene, analyzed the sequence differences between the parents of the gene, and constructed a phylogenetic tree and other preliminary functional verification, which laid the foundation for further in-depth research on the genes controlling the single fruit weight of melons, and greatly accelerated the process of molecular breeding of melons.

## 2. Materials and Methods

### 2.1. Plant Materials and Phenotyping

**Figure 1:**
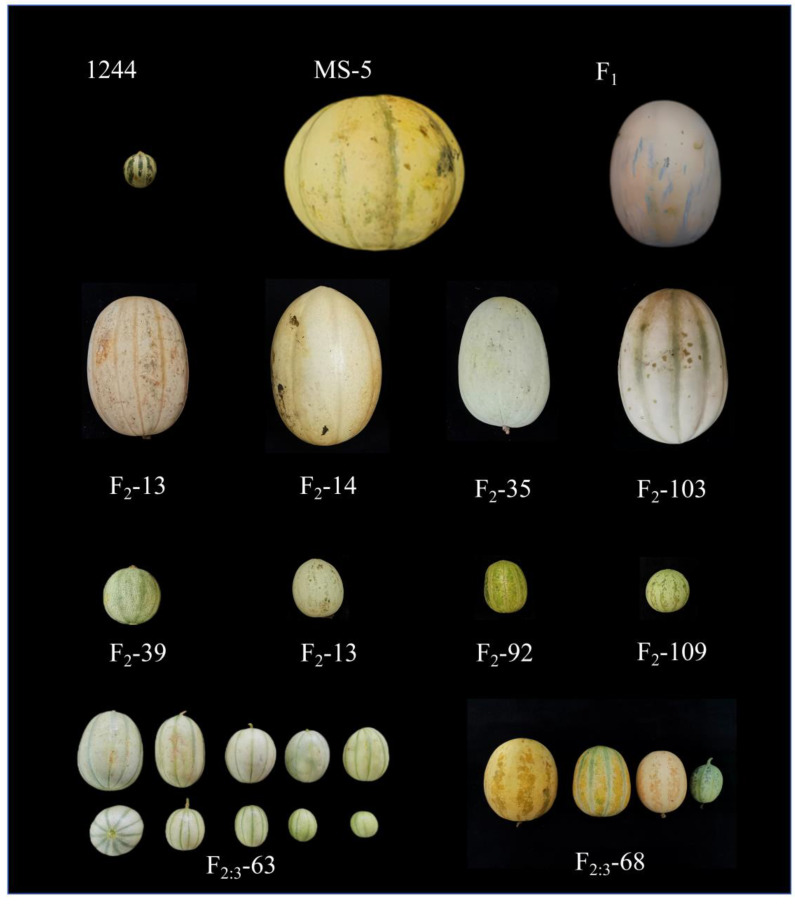
Female parent 1244, male parent MS-5, F_1_, F_2_, and F_2:3_ fruit size variation. Based on the images of the two parental lines and their F_1_ mature fruits, it is important to note the significant differences in fruit weight between the parental melon lines. Images of representative melons of different weights from the F_2_ population and F_2:3_ family lines, all showed differences in fruit size of plants within the F_2_ population and in fruit size of plants of the F_2:3_ family.

Wild-type melon 1244 provided by the Zhengzhou Fruit Tree Research Institute (with a single fruit weight of 22.59 g) was used as the female parent, and thick-skinned melon MS-5 provided by the U.S. Department of Agriculture (with a single fruit weight of 814.97 g) was used as the paternal parent. The offspring generation F_1_, F_2_, and F_2:3_ family lines obtained from the configured hybrid combinations were used as test materials (Figure 1). In the summer (June–September) of 2020, 15 individual 1244, MS-5, and F_1_ plants and 115 F_2_ population plants were used for construction of the specific-locus amplified fragment sequencing (SLAF-seq) genetic maps; for precise mapping and further location of the primary candidate QTL region, 300 F_2:3_ families plants were analyzed in Heilongjiang in the greenhouses at the Heilongjiang Bayi Agricultural University station (latitude 45°46′–46°55′ N) in Summer (June–September) of 2021, and in Autumn (October–December) of 2020 in Sanya, Hainan (latitude 18°09′–18°37′ N), respectively. The melon cultivation methods used included conventional water and fertilizer management, single-plant pollination, and double-vine branching.

### 2.2. Phenotype Collection

The single fruit weight was measured by a balance when the fruits turned color and ripened. From the 1244, MS-5, and F_1_ populations, 3 replicates from 5 single plants were collected and measured. The average single fruit weight of each F_2_ individual was measured in 2–3 fruits per plant, and 20 fruits were measured for each F_2:3_ family member (20 plants per family line, 1 fruit per plant). The average data were recorded, and the genetic patterns of single fruit weight were analyzed.

### 2.3. SLAF-Seq and Indel Molecular Marker Screening

Wang et al. [35] previously performed SLAF-seq (specific length amplified fragment sequencing) using the indicated parent lines and 115 F_2_ plants and obtained candidate regions controlling single fruit weight traits in melon based on the constructed genetic linkage maps. Raw data of SLAF-seq were deposited in the NCBl Sequence ReacArchive (SRA) database under BioProject ID: PRJNA1150761. On this basis, the resequencing data of the parental lines were compared to identify the differences in genome sequence between the parents, and 28 pairs of Indel primers between parents were designed using the software Primer 5.0. Of these, 7 pairs of Indel primers with polymorphisms between parents (Table 1) were used for further localization analysis of the genes responsible for single fruit weight in 300 F_2:3_ families (20 plants per line, 1 fruit per plant) planted in 2020 and 2021, respectively. DNA extraction was performed by the CTAB method. The concentration of the extracted DNA was determined using a nucleic acid protein concentration meter, and diluted to 50 ng/μL for subsequent experiments. The PCR mixture consisted of a total of 10 μL: 1 μL of upstream primer, 1 μL of downstream primer, 3 μL of Taq Master Mix, 1 μL of the DNA template, and 4 μL of ddH_2_O [36]. The PCR (polymerase chain reaction) amplification procedure was as follows: pre-denaturation at 95 °C for 5 min; denaturation at 95 °C for 30 s; annealing at 55 °C for 30 s; extension at 72 °C for 45 s, 35 cycles; extension at 72 °C for 10 min; and storage at 4 °C for subsequent experiments.

### 2.4. Candidate Gene Screening

After identifying candidate regions on the basis of the screened internal markers, candidate genes were detected using the melon genome (DHL92) v3.6.1 (http://cucurbitgenomics.org/) accessed on 23 November 2021, and functional annotations of the candidate genes were found using information from the Gene Ontology (GO) (http://www.geneontology.org) database accessed on 24 November 2021. This information was used to perform functional analyses of genes related to the control of single fruit weight in melon [37,38].

### 2.5. Candidate Gene qRT—PCR Verification

Fruits of 1244 and MS-5 were selected for qRT-PCR validation at 15d, 25d, and 35d days after pollination. RNA extraction for the real-time fluorescence quantitative PCR assay was performed according to the TRIzol method [39]. The RNA was reverse transcribed into cDNA using a Toyo Spun Reverse Transcription Kit, and the qRT—PCR test primers were designed according to the candidate genes, which were synthesized by Shanghai Bioengineering Technology Co. The qRT—PCR mixture was 10 μL in volume and included 0.75 μL of each upstream and downstream primer, 5 μL of SYBR enzyme, 1 μL of cDNA template, and 2.5 μL of ddH_2_O. The PCR amplification procedure was as follows: pre-denaturation at 95 °C for 10 min; denaturation at 95 °C for 15 s; renaturation at 57.5 °C for 30 s; extension at 72 °C for 40 s, 39 cycles, extension at 65 °C for 5 min; and storage at 4 °C for subsequent qRT—PCR verification.

Sequence differences between the parents of the candidate genes were analyzed via DNAMAN 6.0 software. Phylogenetic trees were constructed using the neighbor—joining method to infer evolutionary history. Evolutionary trees were drawn to scale, and evolutionary distances were calculated using the p-distance method in units of the number of amino acid differences at each locus. All positions with less than 50% site coverage were excluded. Evolutionary analyses were conducted in MEGA 7.0.

### 2.6. Data Analysis

#### 2.6.1. Field Experiment

The single fruit weight data of fruits obtained from the field survey were tested for a normal distribution using Microsoft Excel 2021 software, and a histogram of the frequency distribution of the single fruit weight of melons was plotted [40]; statistical analyses of the mean ± standard deviation, maximum, minimum, and extreme deviation of single fruit weight were carried out using Oringe software 2019 [41].

#### 2.6.2. Linkage Map Construction and QTL Analysis

The genetic linkage analysis of polymorphic molecular markers screened in the F_2:3_ family lines was performed using JoinMap 4.0 mapping software, and genetic linkage maps were generated. QTLIciMappingv4.2 software was used for the analysis, and the composite interval mapping (CIM) method was used to locate the QTLs controlling the single fruit weight trait in melon [42]. LOD thresholds were used to evaluate the statistical significance of each QTL and were set using a 1000 permutation test (PT). First, LOD thresholds corresponding to the 0.99 confidence level were considered. If there were no mapped regions, the 0.95 and 0.90 confidence level LOD thresholds were considered [43]. If the QTL interval remained undetected, the PT result was manually lowered to 3.0. The QTLs were named after the trait abbreviation in English + chromosome number.

#### 2.6.3. qRT-PCR Data Analysis

The qRT-PCR assay was completed based on three sample replicates, and the average of the three replicates was calculated using the 2^−ΔΔCt^ method to obtain the relative expression levels of the genes [35].

## 3. Results

### 3.1. Analysis of Single Fruit Weight

Single fruit weights were collected from 1244, MS-5 and F_1_ individuals, the F_2_ population, 2020 and 2021 F_2:3_ families lines and analyzed via Excel software 2019. As shown in the Table 2, there was significant variability in single fruit weight between strains 1244 and MS-5, with mean values of 22.59 ± 11.74 g and 666.78 ± 71.44 g, respectively. In addition, the F_1_ single fruit weight was 160.17 ± 30.06 g, the F_2_ single fruit weight was 100.89 ± 71.97 g, and the single fruit weight of the 2020 and 2021 F_2:3_ families line was 112.29 ± 58.39 g and 106.37 ± 63.14 g. There were significant within-group differences in single fruit weights within the F_2_ population, 2020 and 2021 F_2:3_ family lines, with the mean values ranging between those of 1244 and MS-5.

**Figure 2:**
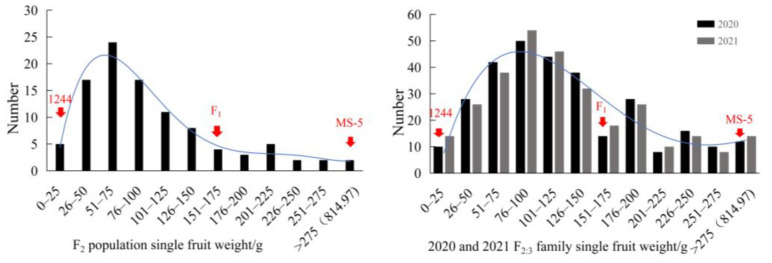
F_2_, 2020 and 2021 F_2:3_ families melon single fruit weight frequency distribution diagram. In this study, we collected data on single fruit weight of the F_2_ population, 2020 and 2021 F_2:3_ families from the cross 1244 × MS-5, and plotted the performance of single fruit weight traits of the two parents and their F_1_ fruits, as well as the frequency distribution of the F_2_ population, 2020 and 2021 F_2:3_ families.

A field survey was conducted and the data were analyzed to evaluate the single fruit weight trait. The frequency distributions for the single fruit weight trait in the F_2_ population, 2020 and 2021 F_2:3_ families lines exhibited wide genetic variation, and a skewed normal unimodal distribution was observed (Figure 2), which indicated that this trait was a quantitative trait controlled by polygenic inheritance.

### 3.2. SLAF Sequencing and QTL Analysis of Melon Single Fruit Weight in the F_2_ Population

**Figure 3:**
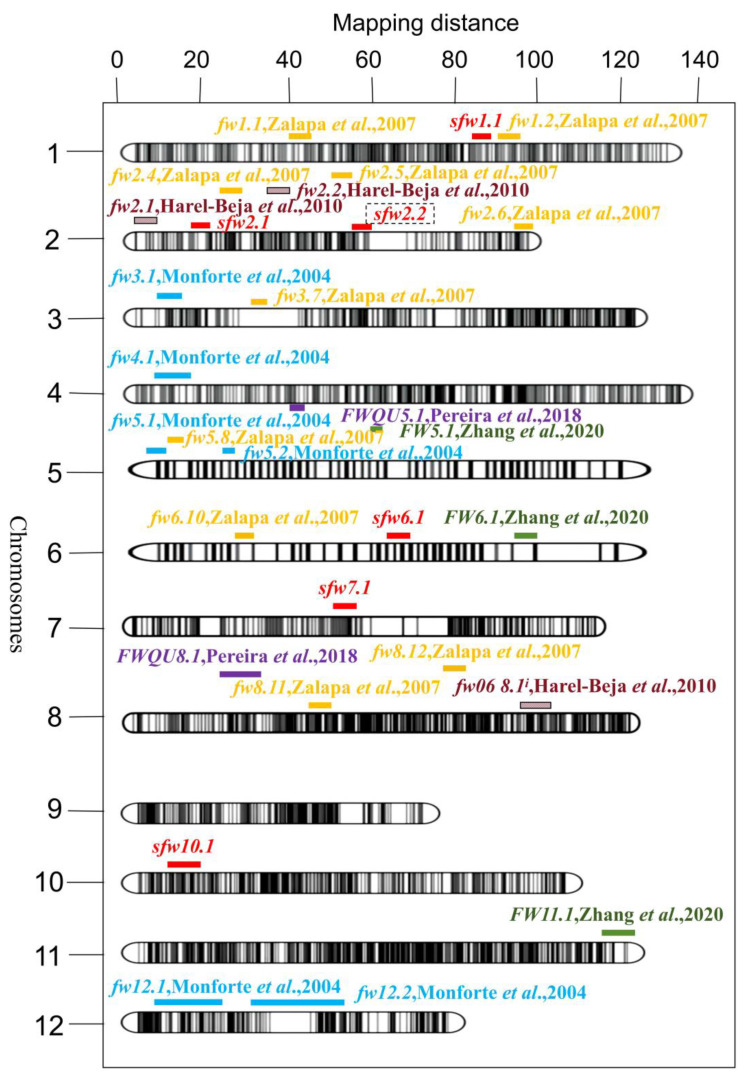
Linkage map and chromosomal locations of QTL for single fruit weight. QTL localization of single fruit weight (sfw) in melon based on experimental data from F_2:3_ family lines. Previous studies of sfw are also listed to compare the loci. The number above the 12 chromosomes represents the length of the map, measured in millimeters (cM). The original QTL names from various publications are used. Six QTLs related to single fruit weight were identified in this study, represented in red bar. The brown bar sfw QTL comes from Harel et al. (2010) [44]; the yellow bar of sfw QTL comes from Zalapa et al. (2007) [45]; the blue bar of sfw QTL comes from Monforte et al. (2004) [46]; the purple bar of sfw QTL comes from Pereira et al. (2018) [27]; and the green bar of sfw QTL comes from Zhang et al. (2020) [47].

A total of 83.12 Gb of data were obtained from SLAF sequencing of the parents and 115 F_2_ plants according to Wang et al. (2021) [35] in the same research laboratory in their study on QTL analysis of flowering-related traits in melon, and a genetic map containing 12 linkage groups was constructed (Figure 3).

Based on the SLAF data, a genetic map containing a total of 12 chromosomes was constructed, with a total length of 1383.88 cM and a genetic spacing of 0.13 cM between markers on the map. A total of six QTLs related to the single fruit weight, *sfw1.1*, *sfw2.1*, *sfw2.2*, *sfw6.1*, *sfw7.1*, and *sfw10.1*, were found in the F_2_ population (shown in Figure 3). The *sfw6.1* locus had a higher LOD value of 4.2, an additive effect of −21.93, a phenotypic contribution of 10.2%, and was located at 30,841,187–38,291,645; this locus contained the most genes, with 354 genes. For *sfw2.2*, both the LOD value and phenotypic contribution rates were the largest among all the loci, at 5.6 and 17.0%, respectively, the additive effect was −45.10, and the interval included 15 genes. Because the *sfw2.2* locus had the highest phenotypic contribution and LOD, the interval of the *sfw2.2* locus was considered a strong candidate interval for controlling the single fruit weight trait in melon and was subjected to further gene localization, as shown in Table 3.

**Figure 4:**
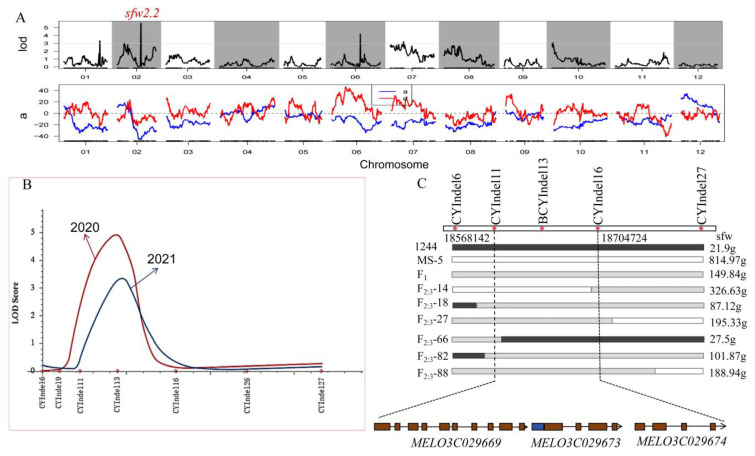
(**A**) Distribution curve of LOD value of QTL loci for single fruit weight trait of melon. The upper part is the distribution curve of the LOD value of the six QTL loci for the single fruit weight trait located, and the lower part is the additive effect (blue line) and dominant effect (red) curve corresponding to the position of the upper part. The *sfw2.2* locus highlighted in red is the main effect QTL locus. (**B**) LOD profile of the primary QTL *sfw2.2* detected in F_2:3_ family lines controlling single fruit weight trait in melon. The red curve represents the QTL positioning for 2020, and the blue curve represents the QTL positioning for 2021. Pink markers on the horizontal axis define the 3.0 LOD interval for each QTL. Fine localization using 300 plants from the F_2:3_ family line designated the 136.5kb region between CYIndel11 and CYIndel6 as the candidate gene region. (**C**) Black coding represents the genotype “1244”, white coding represents the genotype “MS-5”, and striped coding represents the heterozygous genotype. Three coding genes predicted in the 136.5 kb region, and the boxes represent genes.

As shown in Figure 4A, one of the six QTLs controlling the trait of single fruit weight in melon had a LOD value of 3.0, and the remaining five had LOD values greater than 3.0, of which the *sfw2.2* locus located on the second chromosome had the largest LOD value, at 5.6. The dominant effects of the *sfw6.1* and *sfw7.1* loci were positive, and the dominant effects of the *sfw1.1*, *sfw2.1*, *sfw2.2*, and *sfw10.1* loci were all negative. The *sfw2.1* locus had a positive additive effect, and the remaining loci had negative additive effects. The results showed that most of these loci were affected mainly by paternal genetic effects.

### 3.3. Further Localization of the sfw2.2 Locus

Based on the QTL localization of the SLAF sequencing results, the primary candidate intervals controlling the single fruit weight trait in melon were obtained, and further localization was carried out by PCR amplification of 300 F_2:3_ family lines; each family contained 20 individuals grown in both 2020 and 2021 using seven pairs of Indel primers with polymorphisms in the candidate interval. QTL localization of plants from both years revealed that the primary effector was QTL *sfw2.2*, located on chromosome 2 (Figure 4B) within the candidate interval of the Indel markers CYInDel11 and CYInDel16. The LODs were 4.91 and 3.26, contributing 20.75% and 24.78%, respectively, with additive effects of −48.56 and −34.15.

Moreover, we genotyped six key recombinant plants, using A, B, and H to represent the 1244 allele genotype, MS-5 allele genotype, and heterozygous genotype, respectively. The single fruit weight of the recombinant plant F_2:3_-14 was 326.63 g, and the phenotype was B. Analysis of the CYInDel6-CYInDel16 allele of F_2:3_-14, the left fragment was derived from MS-5, and the right fragment was heterozygous for the CYInDel16-CYInDel27 allele. The phenotype and genotype of the recombinant F_2:3_-14 plants were used to determine the right edge of the CYInDel16 target region. Recombinant plant F_2:3_-66 had a single fruit weight of 27.5 g and phenotype A. Based on the CYInDel11-CYInDel27 allele in F_2:3_-66, it was suggested that the right fragment was derived from 1244 and that the left fragment was heterozygous for the CYInDel6-CYInDel11 allele. The phenotype and genotype of the recombinant plant F_2:3_-66 determined the left edge of the CYInDel11 target region. Eventually, the *sfw2.2* locus, which controls single fruit weight in melon, was finely localized to CYInDel11-CYInDel16 at a physical distance of 136.5 kb (Figure 4C).

### 3.4. Candidate Genes and Preliminary Functional Validation

Within the candidate interval of the Indel markers CYInDel11-CYInDel16, three functionally annotated candidate genes in the melon genome were identified through the Cucurbit Genome Database (Melon [DHL92] genome 3.6.1). The annotation information of the candidate genes is shown in Table S1.

**Figure 5:**
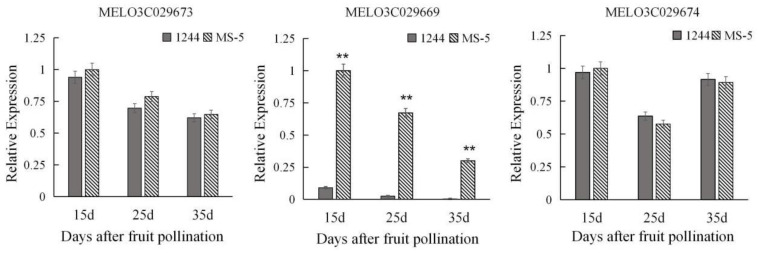
Expression levels of the three candidate genes in fruits of 1244 and MS-5 at different times after pollination. Three genes were quantified using the 2^−ΔΔCT^ method. For the two parents, the expression level of the respective genes in 15d, 25d, and 35d after pollination, respectively. Each sampling was repeated three times, and 10 plants were mixed in equal amounts to form one replicate in two parents. ** indicates an extremely significant difference, *p* < 0.01.

As shown in Figure 5, there was no significant difference between 1244 and MS-5 in the expression of the MELO3C029673 gene or the MELO3C029674 gene at 15d, 25d, and 35d of fruit growth. In contrast, the expression of the MELO3C029669 gene significantly differed between 1244 and MS-5 at 15d, 25d, and 35d of fruit growth, and the expression of this gene was always greater in MS-5 than in 1244. Similarly, the expression of MELO3C029669 gradually decreased in MS-5 with fruit growth and development. And there are multiple base differences between the sequences of the gene’s parents (Figure S1A). Figure S1B shows the evolutionary relationships of MELO3C029669 to homologous genes in other species, with the closest affinity to alfalfa and the furthest to *C melo* var. *makuwa*. In summary, MELO3C029669 was hypothesized to be a candidate gene for controlling single fruit weight in melon.

## 4. Discussion

For crops where the fruit is the product organ, the single fruit weight is an important trait that affects yield and economic efficiency. During fruit growth and development, the fruit weight changes in response to continuous changes in environmental factors and genetic regulation. The study of QTL localization for single fruit weight-related traits in melon and other crops provides a theoretical basis for identifying genes controlling single fruit weight, lays the foundation for plant molecular breeding, and simultaneously improves the yield and efficiency of melon and other crops [48,49,50,51,52,53,54,55,56].

In recent years, extensive research on QTL localization for single fruit weight has been carried out in other cucurbit crops. Kaźmińska et al. [57] located six QTLs controlling the weight of fruits, *fw2.1*, *fw4.1*, *fw10.1*, *fw10.2*, *fw14.1*, and *fw17.1*, in their study on the identification of *Cucurbita maxima* fruit-related QTLs using recombinant selfing lines. *fw4.1* is located on chromosome 4 and was the primary effector QTL, explaining 41.00% and 32.00% of the phenotypic variance in Experiments I and II, respectively. *fw14.1* is located on chromosome 14, and its PVE values are 17.80% and 16.60%, respectively. The remaining QTL for fruit weight was detected in both seasons but with lower PVE values of 10.10–15.40%. Osae et al. [58] crossed the watermelon varieties ZXG1553 and W1-17 to construct an F_2_ population for QTL localization of watermelon fruit size-related traits using CAPS markers. The loci *qFW-3-2*, which controls fruit weight, and *qFL-3-1*, which controls fruit length, were co-located together at 199 cM on chromosome 3, with LOD values of 2.57 and 3.00 and PVE values of 7.03% and 7.73%, respectively. Another QTL for fruit weight, *qFW-3-1*, was located at 36 cM on chromosome 3, with an LOD value of 2.73 and a PVE of 6.63%.

In this study, six QTLs (*sfw1.1*, *sfw2.1*, *sfw2.2*, *sfw6.1*, *sfw7.1*, and *sfw10.1*) associated with the single fruit weight trait of melons were found in the 1st, 2nd, 6th, 7th, and 10th linkage clusters by SLAF sequencing. The LOD value of *sfw2.2* was 5.60, the additive effect was −45.10, and the dominant effect was −13.59; moreover, it had the highest phenotypic contribution rate, at 17%, indicating that this locus is the main effect locus controlling the single fruit weight of melon. Upon identifying the candidate interval of the *sfw2.2* locus, further localization of the *sfw2.2* locus using F_2:3_ family lines planted for two years, and the candidate intervals were shortened so that we could find candidate genes related to single fruit weight. According to current reports, the *FWT1.1* locus localized by Amanullah et al. [21] in 2021 and the *sfw1.1* locus localized in this study are both located on the first chromosome and have very similar genetic distances. The QTL *qFWT6*, detected by Zhang et al. [20] to control FW in melon, and the QTL *sfw6.1*, localized in this study, were close to each other on chr06, although at different locations. Monforte et al. [46] detected six QTLs that control the single fruit weight trait of sweet melons; these loci are different from those in this study and are located on the 3rd, 4th, 5th, and 12th linkage groups, *sfw3.1*, *sfw4.1*, *sfw5.1*, *sfw5.2*, *sfw12.1*, and *sfw12.2*, respectively. Among them, *sfw5.2* had the largest LOD value of 5.99, *sfw4.1* had the smallest LOD value of 2.29, and the range of R^2^ was 0.08–0.34. Santo et al. [59] detected a different QTL locus from the present study *FWQW7.1* in a study on the fruit morphology and ripening-related QTLs in a newly developed introgression line collection of the elite varieties ‘Védrantais’ and ‘Piel de Sapo’. It can be used in melon breeding programs to change the size of the fruit without affecting the shape of the fruit. Similarly, Zhang et al. [47] identified three QTLs associated with single fruit weight in melon via QTL analyses for traits related to melon fruit. *FW6.1* was found to be located on chromosome 6; the *sfw6.1* locus identified in this study is also located on chromosome 6 but at a different position. The genetic distance of the *FW6.1* locus was 98.01–104.07 cM, the LOD value was 3.93, and the additive effect was 0.10. The *FW5.1* and *FW11.1* loci were located on chromosomes 5 and 11, respectively. *FW5.1* had the highest LOD value of 4.78 with a phenotypic variance of 6.85%, while *FW11.1* had the lowest LOD value of 2.69 with a phenotypic variance of 3.78%. Harel-Beja et al. [44] crossed two melon subspecies, “PI 414723” and “Dulce”, and constructed a recombinant inbred (RI) population to establish a genetic linkage map of melon enriched for fruit traits. Two and one QTL related to the single fruit weight trait of melon were identified on LG2 and LG8 (*fw2.1*, *fw2.2,* and *fw06 8.1^i^*, with LOD values of 8.58, 3.02, and 4.14, respectively). The first two loci, along with *fw2.1* and *fw2.2* identified in this study, are located on chromosome 2 at different genetic distances. QTLs associated with melon fruit length, *fl2.1* and *fl8.1*, were also detected on LG2 and LG8, respectively, with LOD values of 11.80 and 2.99. Thus, the two QTLs for weight and length, *fw2.1* and *fl2.1*, co-segregated and overlapped on LG2 with higher LOD scores. This finding suggests that fruit weight in this melon population is related to fruit length. In the genetic study of traits related to melon yield, Zalapa et al. [45] used molecular markers such as SSR and RAPD (random amplified polymorphic DNA) to detect 12 QTLs related to melon single fruit weight in parental line, RIL (recombinant inbred lines), and to 3 control varieties (Esteem, Sol Dorado, and Hales Best Jumbo), including *fw1.1*, *fw1.2*, *fw2.4*, *fw2.5*, *fw2.6*, *fw3.7*, *fw5.8*, *fw6.10*, *fw8.11*, and *fw8.12*, where the *fw1.2* locus is genetic distance similar to the *fw1.1* locus localized in the first linkage cluster in this study. The LOD values ranged from 3.59 to 12.53, and the additive effects ranged from −0.18 to 0.42. Among them, four loci, *fw5.8*, *fw6.10*, *fw8.11,* and *fw8.12*, were consistently detected in different varieties, laying the foundation for further screening of genes related to fruit weight.

The candidate gene obtained in this study was *MELO3C029669*, which is related to fimbrin-5 (a filamentous protein) according to gene functional annotation (*C melo fimbrin-5* (LOC103495662), transcript variant X3, mRNA) based on the gene ID. In a previous study on *Arabidopsis thaliana*, Zhang et al. [33] described five members of the Fimbrin family, of which *AtFIM4* and *AtFIM5* were expressed at higher levels in pollen than in other tissues. These findings suggest the involvement of *AtFIM4* and *AtFIM5* in the growth and regulation of pollen tubes. Loss of function of *AtFIM5* leads to slow pollen tube growth and pod grain deficiency, which affects pod length, morphology, and fruit set; moreover, simultaneous reductions in *AtFIM4* and *AtFIM5* expression cause even more severe phenotypes, impacting Arabidopsis size and yield at maturity. Based on the results of this study, Ding et al. [60] from Northwestern University further reported that Arabidopsis plants exhibit a series of growth hormone-related phenotypes upon simultaneous deletion of *AtFIM4* and *AtFIM5*. The double mutant *atfim4-1*/*atfim5-2* presented a longer primary root length, more lateral roots, more apical meristematic tissues, and a significant decrease in growth hormone content at the static position of the root tip. Taken together, these findings suggest that the absence of *AtFIM4* and *AtFIM5* may affect the transport of growth hormone in roots and that growth hormones have a major impact on regulating plant size during plant growth and development. From this, it can be deduced that fimbrin-5 also affects the weight of melon plants during growth and development; thus, the *MELO3C029669* gene is a reasonable candidate gene associated with single fruit weight trait in melons.

To further validate the speculation that MELO3C029669 is a candidate gene, DNAMAN software was used to analyze the sequence difference features between the parents of the genes, and a total of 12 base differences in the sequence between the parents were found. Among them, four differences occurred in the base sequence of the parent MS-5, and eight differences occurred in the base sequence of the parent 1244, as shown in Figure S1A. The candidate gene *MELO3C029669* was more conserved in MS-5 and had more variation in 1244. In summary, *MELO3C029669* is likely the most likely candidate gene for controlling the weight of a single fruit in melons.

The candidate gene *MELO3C029669* is related to fimbrin-5 (a filamentous protein) according to gene function annotation. To investigate the evolutionary relationship of this gene to its homologs in other species, the amino acid sequence encoded by the *MELO3C029669* gene was subjected to BLAST in the NCBI online database to obtain the amino acid sequences of the homologous genes in several different plant species, and phylogenetic evolutionary trees were constructed using the neighbor-joining (NJ) method in MEGA 7.0 software (Figure S1B). Different evolutionary branch lengths represent different degrees of evolution; the longer the branch, the greater the degree of evolution, and vice versa. The results showed that the melon single fruit weight candidate gene *MELO3C029669* was most closely related to that in *P sativum*, with the Gene ID KAl5432624.1 and furthest related to *C melo* var. *makuwa*, with the Gene ID TYK04175.1.

## 5. Conclusions

In this study, sfw2.2 was identified as the main QTL locus controlling single fruit weight traits in melons based on the genetic linkage map constructed by the research group previously. On this basis, the QTL of the sfw2.2 locus was further localized between the CYInDel11 marker and the CYInDel16 marker, with LOD values of 4.91 and 3.26, respectively. In the candidate region, 3 candidate genes were identified within a candidate interval of 136.5 Kb. The qRT-PCR results verified that *MELO3C029669* genes were different expressed in melon fruit development, and evolutionary relationships analysis also hypothesized that *MELO3C029669* is a candidate gene for controlling the single fruit weight of melon, which lays the foundation for the next step of gene function validation.

## Figures and Tables

**Table 1:** Information about primers for fine localization.

Number	Primer Name	Genomic Location	Forward Sequence	Reverse Sequence
1	CYIndel6	18,524,772–18,524,791	CCTAAGGCTAAAGGATATCG	AAACATTTTGGAAGGGTAAG
2	CYIndel9	18,560,853–18,561,288	AGTAAAGATGGGGTGAAAAC	TGTGAAAAATGAGGATGGAA
3	CYIndel11	18,568,142–18,568,533	GCTGTAGAACACATTAAGGACGA	TAAAGACCTTGGAAAATCGAATA
4	CYIndel13	18,602,885–18,603,339	CTCCTGCTTGTGTCCTCATG	TCAGTCTTCTCAAATCCTCCC
5	CYIndel16	18,704,309–18,704,724	TCTTTCGGCTTCACTTGCTT	GAAGAGCGTTGTGCTGTGG
6	CYIndel26	18,906,922–18,907,357	TTTGTGAGAGACAAGTTTGAGT	ATACAGGAAGATTTTCTGCTAC
7	CYIndel27	18,920,443–18,920,801	TCCTCGAAGTCGGTGGTAAC	TGGTCCTATCAATGAGGCAAAT

**Table 2:** Phenotypic analysis of single fruit weight traits in melon.

Generation	Mean ± SD		CV	Maximum	Minimum	Range	H^2^ of Trait
1244	22.59 ± 11.74 a		51.97%	43.83	3.83	40.00	52.51%
MS-5	814.97 ± 70.12 b		8.60%	931.84	682.72	249.12	
F_1_	160.17 ± 30.06		18.77%	201.40	99.15	102.25	
F_2_	100.89 ± 71.97	Max: 170.99 ± 72.60 a	71.34%	406.80	17.40	389.40	
		Min: 59.72 ± 23.57 b					
F_2:3-2020_	106.37 ± 63.14	Max: 163.42 ± 67.33 a	59.36%	395.70	18.69	377.01	
		Min: 65.32 ± 25.81 b					
F_2:3-2021_	112.29 ± 58.39	Max: 151.60 ± 55.24 a	52.00%	326.63	19.46	307.17	
		Min: 70.52 ± 20.13 b					

Note: *p* < 0.05. SD is standard deviation. CV values are coefficients of variation and indicate the degree of variability. H^2^ indicates heritability.

**Table 3:** QTL loci for single fruit weight traits by F_2_ population in melon.

Trait	QTLsite	Linkage Group	Genome Start Position	Genome End Position	LOD	ADD	PVE (%)	Gene Number
Single fruit weight	*sfw1.1*	1	34,295,745	34,435,727	3.30	−28.38	8.30	17
	*sfw2.1*	2	1,626,517	1,635,504	3.00	17.81	3.10	1
	*sfw2.2*	2	18,344,184	18,881,371	5.60	−45.10	17.00	15
	*sfw6.1*	6	30,841,187	38,291,645	3.20	−21.93	8.60	354
	*sfw7.1*	7	4,758,046	7,547,788	3.20	−25.56	5.90	113
	*sfw10.1*	10	485,913	504,601	3.10	−30.64	8.30	2

LOD, logarithm of the odds; ADD, additive effect; PVE, phenotypic variation explained by specific QTL of total variances.

## Data Availability

Data are contained within the article and Supplementary Materials.

## Supplementary Materials

The following supporting information can be downloaded at: https://www.mdpi.com/article/10.3390/genes15091138/s1, Figure S1: Sequence comparisons between parents of candidate genes and analysis of the evolutionary relationships in other species. A. The candidate gene is located in chr02 with genomic location 18,564,512–18,570,079. DNAMAN 1 is the parent and DNAMAN 2 is the maternal parent, and the light blue markers in the sequence comparison plot indicate sequence differences between the parents. B. A phylogenetic tree was constructed with MEGA 7.0 using the rootless neighbor-joining (NJ) method. The tree was drawn to scale and analyzed involving 10 amino acid sequences. The different evolutionary branch lengths represent the degree of evolutionary branch changes, with longer representing greater gene changes and shorter representing smaller gene changes. The numbers on the branches are greater than 70%, indicating that the reliability of the constructed evolutionary tree is good. Table S1: Candidate gene annotation information.
