# Supplementary material for: Multiple Localization Analysis of the Major QTL—sfw 2.2 for Controlling Single Fruit Weight Traits in Melon Based on SLAF Sequencing
Source: Genes (Basel). 2024 Aug 28;15(9):1138. doi: 10.3390/genes15091138 (PMC11430989; doi:10.3390/genes15091138)
Supplement: Supplementary file 1 [file genes-15-01138-s001.zip › Supplementary materials/Figure S1.pdf]

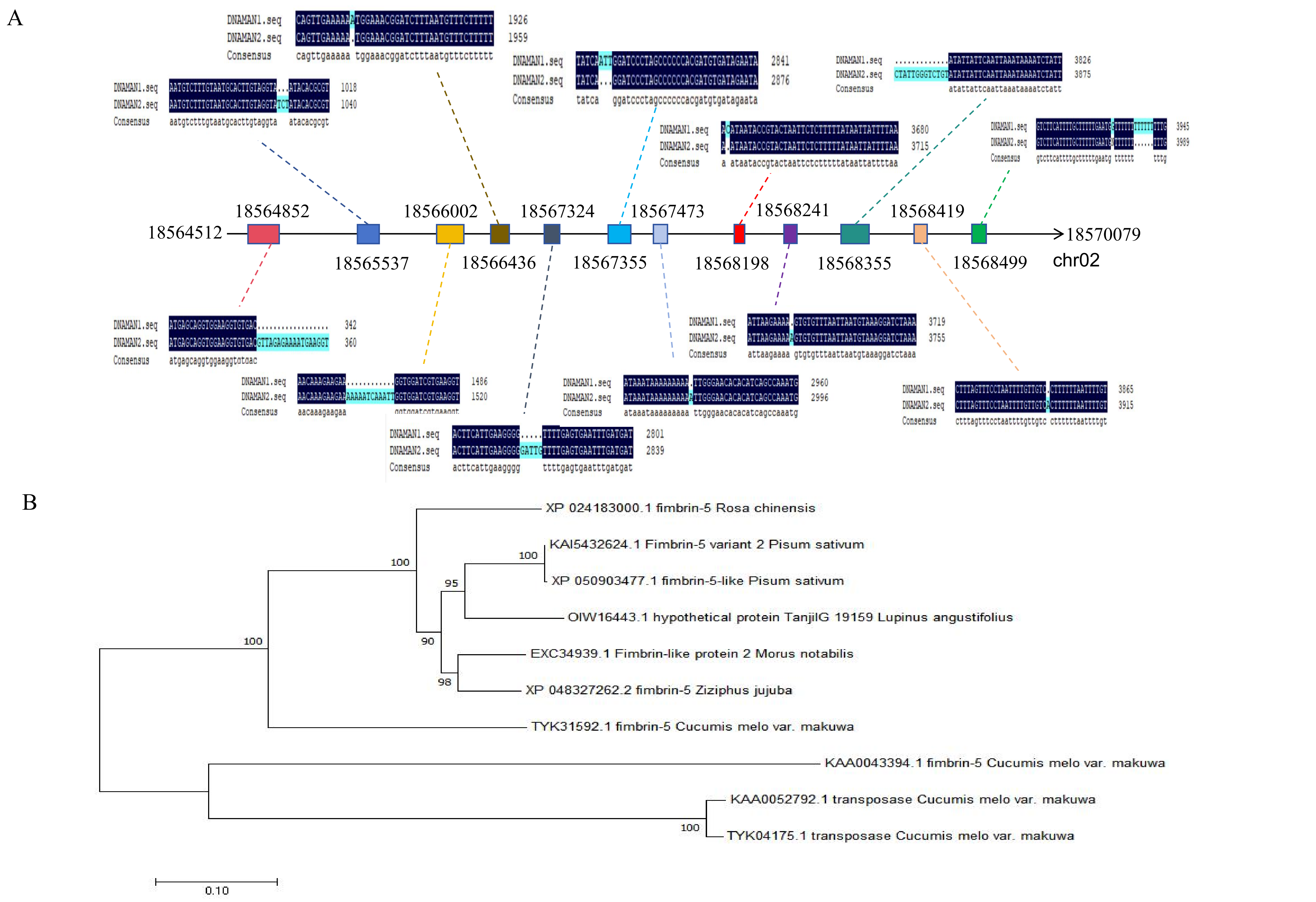

FigureS1 A. Sequence comparisons between parents of candidate genes. The candidate gene is located in chr02 with genomic location 18564512-18570079, DNAMAN 1 is the parent and DNAMAN 2 is the maternal parent, and the light blue markers in the sequence comparison plot indicate sequence differences between the parents. Different colour markers on the chromosomes indicate multiple sequence differences between the parents within the candidate gene interval. B. Analysis of the evolutionary relationships of the candidate gene MELO3C029669 in other species. A phylogenetic tree was constructed with MEGA 7.0 using the rootless neighbour joining (NJ) method. The tree was drawn to scale and analysed involving 10 amino acid sequences. The different evolutionary branch lengths represent the degree of evolutionary branch changes, with longer representing greater gene changes and shorter representing smaller gene changes. The numbers on the branches are greater than 70%, indicating that the reliability of the constructed evolutionary tree is good.
